# Injury morbidity in an urban and a rural area in Tanzania: an epidemiological survey

**DOI:** 10.1186/1471-2458-5-11

**Published:** 2005-01-28

**Authors:** Candida Moshiro, Ivar Heuch, Anne Nordrehaug Åstrøm, Philip Setel, Yusuf Hemed, Gunnar Kvåle

**Affiliations:** 1 Centre for International Health, University of Bergen, Norway; 2 Muhimbili University College of Health Sciences, Dar es Salaam, Tanzania; 3 Department of Mathematics, University of Bergen, Norway; 4 MEASURE Evaluation, Carolina Population Center, University of North Carolina at Chapel Hill, USA; 5 Adult Morbidity and Mortality Project and Tanzanian Ministry of Health, Tanzania

## Abstract

**Background:**

Injuries are becoming a major health problem in developing countries. Few population based studies have been carried out in African countries. We examined the pattern of nonfatal injuries and associated risk factors in an urban and rural setting of Tanzania.

**Methods:**

A population-based household survey was conducted in 2002. Participants were selected by cluster sampling. A total of 8,188 urban and 7,035 rural residents of all ages participated in the survey. All injuries reported among all household members in the year preceding the interview and resulting in one or more days of restricted activity were included in the analyis.

**Results:**

A total of 206 (2.5%) and 303 (4.3%) persons reported to have been injured in the urban and rural area respectively. Although the overall incidence was higher in the rural area, the incidence of major injuries (≥ 30 disability days) was similar in both areas. Males were at a higher risk of having an injury than females. Rural residents were more likely to experience injuries due to falls (OR = 1.6; 95% CI = 1.1 – 2.3) and cuts (OR = 4.3; 95% CI = 3.0 – 6.2) but had a lower risk of transport injuries. The most common causes of injury in the urban area were transport injuries and falls. In the rural area, cuts and stabs, of which two thirds were related to agriculture, formed the most common cause. Age was an important risk factor for certain types of injuries. Poverty levels were not significantly associated with experiencing a nonfatal injury.

**Conclusion:**

The patterns of injury differ in urban and rural areas partly as a reflection of livelihoods and infrastructure. Rural residents are at a higher overall injury risk than urban residents. This may be important in the development of injury prevention strategies.

## Background

Injuries have been recognised as a major public health problem in both developed and less developed countries [1]. It is generally acknowledged that this problem is growing rapidly in sub-Saharan Africa [2]. Studies in Tanzania show that injuries are an important cause of death among adults [3], and accounted for 12% of all admissions at the national hospital in the country's largest city [4].

Studies on the magnitude of injuries and the groups at risk, have been conducted world-wide, and especially in developed countries. Hospital based studies, which are commonly reported from developing countries, presumably provide a representative picture of the prevalence and incidence of serious injury, but only a partial picture of the circumstances in which injuries occur. Given the limited access to hospital care in poor countries, however, data based on health facility data are not likely to be representative. In contrast, population-based studies are costly and rarely carried out particularly on topics such as injury, which are not high on the public health agenda in developing countries at present.

Several studies have been conducted in high-income countries to examine factors associated with injury morbidity [5-7]. In developing countries, a number of population-based studies on nonfatal injuries have been done [8-14]. In order to understand the circumstances and risk factors associated with nonfatal injuries, we conducted a community-based study in an urban and a rural location of Tanzania. We describe injury patterns in both settings. We also investigate demographic and socioeconomic factors associated with nonfatal injuries.

## Methods

### Study area

The survey was conducted in Dar es Salaam city (an urban area) and Hai District (a rural area). These areas are part of a health and demographic surveillance system carried out by the Adult Morbidity and Mortality Project (AMMP) in six districts in Tanzania from 1992 to 2004. One aim of the project was to measure rates and causes of morbidity and mortality. At the time this investigation was carried out, the areas were being prospectively monitored through repeated censuses to ascertain the resident population at risk. Deaths were recorded through an active reporting system and probable cause of death determined by a validated verbal autopsy [15,16].

Dar es Salaam city lies on the east coast of Tanzania. The AMMP demographic surveillance population was situated in two of the city's three municipalities. These areas contained 8 'branches' (an urban administrative unit) and covered 63,330 persons in 15,269 households living in urban and peri-urban neighbourhoods. Hai District lies on the South-Western slopes of Mount Kilimanjaro in Northern Tanzania. The AMMP demographic surveillance area in Hai covered 51 out of 61 villages in the district and around 62% of the total district population (159,906 persons in 40,238 households). Agriculture, livestock keeping and commercial mining are the main economic activities there. Details of the study population have been described elsewhere [15,16].

### Sampling procedure

In the urban surveillance area, an initial cluster sample of 500 households was randomly selected. Because initial data collection yielded fewer injuries than expected in the first two enumeration areas, the sample size was increased to 2,000 households with the difference made up of households selected at random from the remaining six surveillance branches. Thus, the final sample under-represented the first two branches. Information was sought on all individuals residing in the selected households. A two-stage cluster sampling method was adopted in selecting the rural sample. In the first stage, using existing AMMP data on mortality and poverty, six out of 51 villages were selected to represent different levels of socio-economic status and injury mortality. A random sample of 2,000 households was obtained from the selected villages in the second stage. All individuals in the selected households were included in the survey.

### Ethical clearance and informed consent

Ethical clearance for this study was given by the Tanzania Commission for Science and Technology and the Regional Committee for Medical Research Ethics in Norway. Informed verbal consent was sought from each family. For children below age 15, parents or guardians were interviewed; for adolescents aged 15 to 18, consent was obtained from both the parent and the child.

### Data collection

The survey tool was translated into Swahili, back translated into English, and pre-tested before use in the field. Two questionnaires were used in the study. Questionnaire 1 was a screening form used to identify whether a household member had an injury in the past one year that resulted in losing one or more days of 'normal' activity (e.g. not being able to work or go to school). The head of household or any other responsible person was interviewed to obtain information about the household members. Variables included were age, sex, relationship to head of household, level of education, religion, marital status and occupation.

Questionnaire 2 was used to record the circumstances in which the injury occurred. Some of the variables included were: month and year when the injury occurred; cause of the injury; place of occurrence; activity at time of injury; length of disability; and health facility use. Efforts were made to interview the injured person if an adult, otherwise we interviewed an informed member of the injured person's household. The number of days with restricted activity (disability days) was considered as a measure of severity of injury. Poverty was assessed at the household level using data from the 2000–2001 National Household Budget Survey and variables from AMMP data. The measure of poverty used was a predicted value of monthly consumption expenditure per adult equivalent for each household included in the study [17,18].

### Statistical analysis

Data analysis was done using STATA (version 7). Bivariate analyses were performed by cross tabulations and the chi-squared test was used to test for homogeneity. In the case of multiple injuries, the most recent injury episode was considered in all the analysis. Multiple logistic regression was used to examine the influence of socio-demographic and socio-economic factors on the risk of being injured, controlling for potential confounding variables. Odds ratios are reported with 95% confidence intervals. Tests for trend in associations over groups defined by other factors were performed where appropriate.

Preliminary analysis indicated that a long recall period underestimated annual injury rates, with the effect being greater for injuries resulting in <30 disability days while the rates for injuries resulting in 30 or more disability days were quite stable[19]. We therefore categorized severity of injury as 'minor' if resulting in less than 30 days of lost activity and 'major' if resulting in 30 or more days of lost activity. This kind of categorization has also been used in the Ghana study [13]. The category for major injuries is less likely to include actual minor injuries, and therefore constitutes a well-defined small group. However, the category for minor injuries might include some injuries that were actually severe. About 37 (7.3%) individuals who sustained an injury reported between 15 and 21 disability days, with only 3 reporting 22 to 29 days of restricted activity. Poverty quintiles were classified as most poor, very poor, poor, less poor, or least poor in terms of socioeconomic status. Adjustment for clustering was performed with standard STATA commands for analysis of survey data.

## Results

Data were gathered on a total of 15,223 individuals residing in 3,653 households. The urban sample included 8,188 individuals while the rural sample included 7,035 persons. The response rates were 89% and 92% for the urban and rural areas respectively. The rural sample had a larger proportion of individuals aged 44 years and above (24%) compared to the urban area (11%). This was comparable to national averages as reported by the 2002 national census in Hai (17%) and Dar es Salaam (10%)[20]. Educational status was higher in the urban area.

Of the total sample, 509 persons reported to have sustained an injury in the past one year preceding the survey, representing an injury incidence of 32.7 per 1,000 persons per year (95% CI= 29.9 – 35.7). The incidence for all, minor and major injuries was 24.5, 16.4 and 8.1 per 1,000 persons per year for Dar es Salaam, and 42.5, 32.8 and 9.7 for Hai district. The mean age of the injured was 27.6 years (standard deviation 20) and 62% were males. Almost all injuries were unintentional (96%). On average, 14 days of normal activity were lost per person because of an injury.

In Dar es Salaam, the most common cause of injury reported in both males and females was transport injuries, followed by falls and cuts (Table 1). In Hai, cuts ranked first, followed by falls and transport injuries. The proportion of individuals who sustained transport injuries in the urban area was four times higher than in the rural area (33.0% vs 7.6% respectively; p < 0.001). Cuts and stabs accounted for 49% of the injuries in Hai compared to only 18% in Dar es Salaam (p < 0.001). There was no statistical difference in the distribution of injury categories between males and females in the urban area (p = 0.37). In the rural area, males and females differed with respect to the most common causes of injuries (p < 0.001), with transport injuries experienced almost exclusively by males, and cuts being more frequent in females.

**Table 1:** Cause of nonfatal injury by sex in the urban and rural areas

**Cause of injury**	**Total**		**Males**		**Females**	
			
	**No.**	**%**	**No.**	**%**	**No.**	**%**
***Urban (Dar es Salaam)***						
***Total***	***206***	***100***	***136***	***100***	***70***	***100***
Transport injuries	68	33.0	43	31.6	25	35.7
Falls	56	27.2	35	25.7	21	30.0
Cuts/stabs	38	18.5	26	19.1	12	17.1
Burn	12	5.8	6	4.4	6	8.5
Struck by object	12	5.8	10	7.4	2	2.9
Animal bites	4	1.9	3	2.2	1	1.4
Assault	7	3.4	4	2.9	3	4.3
Other	9	4.4	9	6.7	0	0
						
***Rural (Hai)***						
***Total***	***303***	***100***	***177***	***100***	***126***	***100***
Transport injuries	23	7.6	22	12.4	1	0.8
Falls	83	27.4	49	27.7	34	26.9
Cuts/stabs	149	49.2	77	43.5	72	57.1
Burns	18	5.9	8	4.5	10	7.9
Struck by object	11	3.6	11	6.2	0	0.0
Animal bites	6	1.9	4	2.3	2	1.6
Assault	3	0.9	3	1.7	0	0
Other	10	3.3	9	5.1	1	0.8

**Figure 1:**
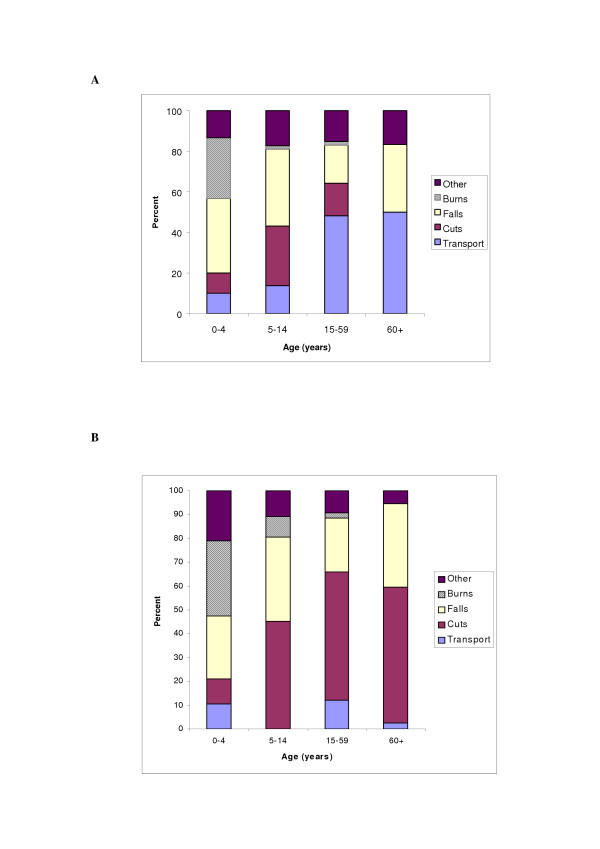
1a: Cause of injury by age in Dar es Salaam (urban area) 1b: Cause of injury by age in Hai (rural area)

As expected, the cause of injury varied by age (Figure 1). In the urban area, transport injuries were most common among adults aged 15 years and above while burns were common among children under 5 years. Cuts and stabs ranked second as a cause of injury among the 5 to 14 year olds. In Hai, cuts were the commonest cause of injury in all age groups except among 0–4 year olds where burns and falls were most frequent.

Major injuries accounted for 33% and 23% of all injuries in Dar es Salaam and Hai respectively (Table 2). The percentage of transport injuries resulting in a major injury was 41% and 30% in the urban and rural areas respectively. Of cuts or stabs in the rural area, 13% were major whilst in the urban area, 21% of the cuts were major. More than one third of the falls were categorised as major injuries in both areas.

**Table 2:** Major injuries as a percentage of all injuries (≥ 30 disability days) by cause and sex in the urban and rural areas

**Cause of injury**	**No. of all injuries**	**Both sexes**	**Males**	**Females**
		
		Percent of major injuries		
	
***Dar es Salaam (Urban)***				
**Total**	**206**	**33.0**	**28.7**	**41.4**
Transport injuries	68	41.2	34.9	52.0
Falls	56	35.7	28.6	47.6
Cuts/stabs	38	21.1	23.1	16.7
Burn	12	33.3	16.7	50.0
Struck by object	12	33.3	30.0	50.0
Other	20	20.0	30.8	0
				
***Hai (Rural)***				
**Total**	**303**	**22.8**	**23.7**	**21.4**
Transport injuries	23	30.4	27.3	100.0
Falls	83	38.6	36.7	41.2
Cuts/stabs	149	12.8	13.0	12.5
Burns	18	16.7	25.0	10.0
Struck by object	11	45.5	45.5	0
Other	19	15.8	10.0	28.6

After controlling for age, sex and education, persons residing in Hai were 1.7 times as likely to have had an injury in the past one year as compared to those residing in Dar es Salaam (Table 3). Males had a higher risk of being injured than females. Those with primary education only had an increased risk of having an injury compared to their counterparts who had no formal education. Children aged 5 to 14 had slightly higher odds of sustaining a minor injury compared to adults, while for major injuries adults aged 45 years and above were at an increased risk (p < 0.01 comparing trends with age for minor and major injuries). After adjusting for age, sex, education and area, there was no significant association between poverty and risk of nonfatal injury. Male sex turned out to be the only significant risk factor for major injuries.

**Table 3:** Adjusted odds ratios (OR) for all, minor (<30 disability days) and major (≥ 30 disability days) injuries by demographic and socio-economic factors

**Factors**	**Total**	**All injuries**		**Major injuries**		**Minor injuries**	
	**Sample**	**No.**	**OR^a ^(95% CI)**	**No.**	**OR^a ^(95% CI)**	**No.**	**OR^a ^(95% CI)**

**Area**							
Dar es Salaam (Urban)	8188	206	1.0	68	1.0	138	1.0
Hai (Rural)	7035	303	1.66 (1.37 – 2.02)	69	1.09 (0.75 – 1.58)	234	1.94 (1.54 – 2.44)
			*p < 0.001*		*p = 0.65*		*p < 0.001*
Age*							
0–4	1720	49	1.28 (0.83 – 1.99)	13	0.85 (0.38 – 1.91)	36	1.53 (0.91 – 2.57)
5–14	3711	140	1.23 (0.98 – 1.56)	29	0.88 (0.56 – 1.40)	111	1.37 (1.04 – 1.79)
15–44	7140	212	1.0	59	1.0	153	1.0
45+	2651	108	1.25 (0.97 – 1.59)	36	1.57 (0.99 – 2.47)	72	1.12 (0.84 – 1.50)
			*p = 0.20*		*p = 0.10*		*p = 0.11*
**Sex**							
Females	7844	196	1.0	56	1.0	140	1.0
Males	7379	313	1.75 (1.46 – 2.12)	81	1.57 (1.11 – 2.21)	232	1.81 (1.46 – 2.25)
			*p < 0.001*		*p < 0.01*		*p < 0.001*
Education**							
None	3546	97	1.0	30	1.0	67	1.0
Primary	9674	362	1.50 (1.09 – 2.06)	92	1.02 (0.59 – 1.74)	270	1.77 (1.21 – 2.59)
Secondary+	2001	50	1.18 (0.77 – 1.82)	15	0.79 (0.37 – 1.71)	35	1.41 (0.84 – 2.36)
			*p = 0.01*		*p = 0.67*		*p < 0.01*
Poverty quintiles (n = 12320)***							
1 (Most poor)	2471	95	1.13 (0.82 – 1.56)	22	1.05 (0.56 – 1.96)	73	1.15 (0.80 – 1.66)
2	2462	78	0.92 (0.66 – 1.29)	21	1.01 (0.52 – 1.97)	57	0.89 (0.61 – 1.32)
3	2466	81	0.96 (0.69 – 1.32)	23	1.11 (0.61 – 2.01)	58	0.91 (0.62 – 1.32)
4	2462	72	0.86 (0.61 – 1.22)	25	1.24 (0.67 – 2.29)	47	0.74 (0.49 – 1.12)
5 (Least poor)	2459	83	1.0	20	1.0	63	1.0
			*p = 0.49*		*p = 0.96*		*p = 0.19*

* 1 missing subject, ** 2 missing subjects

^a^Adjusted odds ratios from logistic regression models that included all variables in table except poverty

*** Adjusted for age, sex, area and education

p-value for test of heterogeneity

We investigated associations between different factors and some of the most frequent causes of injury (Table 4). Rural residents were significantly less likely to have transport injuries compared to urban dwellers (OR = 0.39; 95% CI 0.23 – 0.66). Children aged between 5 and 14 years were less likely to sustain transport injuries compared to adults aged 15–44 years. Males had a significantly increased risk of having transport injuries compared to females. However, household consumption expenditure was not associated with risk of transport injuries. In the rural area, the commonest type of transport involved was bicycle (52%) while in the urban area cars and trucks (46%) and commercial buses (22%) were frequently involved (Table 5). In Dar es Salaam, 41% of those involved in transport injuries were pedestrians who were struck by motor vehicles or bicycles, whereas in Hai, the largest proportion of those with a transport injury were vehicle occupants (52%) followed by cyclists (35%).

**Table 4:** Adjusted odds ratios (OR) for transport injuries, falls and cuts or stabs by demographic and socio-economic factors

**Factors**	**Total Sample**	**Transport injuries**		**Falls**		**Cuts/stabs**	
		**No.**	**OR (95% CI)^a^**	**No.**	**OR (95% CI)^a^**	**No.**	**OR (95% CI)^a^**

**Area**							
Dar es Salaam (Urban)	8188	68	1.0	56	1.0	38	1.0
Hai (Rural)	7035	23	0.39 (0.23 – 0.66)	83	1.56 (1.09 – 2.25)	149	4.27 (2.96 – 6.15)
			*p < 0.001*		*p = 0.01*		*p < 0.001*
Age*							
0–4	1720	5	0.52 (0.14 – 1.97)	15	2.21 (0.98 – 4.98)	5	0.35 (0.12 – 0.99)
5–14	3711	8	0.28 (0.13 – 0.63)	51	2.44 (1.58 – 3.79)	54	1.12 (0.77 – 1.62)
15–44	7140	60	1.0	41	1.0	80	1.0
45+	2651	18	1.01 (0.56 – 1.83)	32	1.98 (1.22 – 3.21)	48	1.19 (0.82 – 1.74)
			*p < 0.01*		*p < 0.001*		*p = 0.07*
**Sex**							
Females	7844	26	1.0	55	1.0	84	1.0
Males	7379	65	2.66 (1.64 – 4.29)	84	1.62 (1.14 – 2.30)	103	1.38 (1.03 – 1.85)
			*p < 0.001*		*p < 0.01*		*p = 0.03*
Education**							
None	3546	10	1.0	30	1.0	23	1.0
Primary	9674	67	1.76 (0.63 – 4.93)	96	1.56 (0.90 – 2.69)	151	1.61 (0.98 – 2.66)
Secondary+	2001	14	1.10 (0.34 – 3.56)	13	1.50 (0.67 – 3.38)	13	1.03 (0.48 – 2.21)
			*p = 0.15*		*p = 0.27*		*p = 0.05*
Poverty quintiles (n = 12320)***							
1 (Most poor)	2471	15	1.18 (0.56 – 2.49)	19	0.67 (0.36 – 1.22)	41	1.36 (0.81 – 2.28)
2	2462	12	0.90 (0.41 – 2.01)	24	0.85 (0.48 – 1.51)	35	1.14 (0.68 – 1.91)
3	2466	12	0.93 (0.43 – 2.01)	24	0.84 (0.48 – 1.49)	27	0.87 (0.51 – 1.48)
4	2462	13	0.99 (0.47 – 2.11)	21	0.76 (0.41 – 1.39)	26	0.86 (0.50 – 1.50)
5 (Least poor)	2459	14	1.0	27	1.0	30	1.0
			*p = 0.96*		*p = 0.74*		*p = 0.31*

* 1 missing subject, ** 2 missing subjects

^a^Adjusted odds ratios from logistic regression models that included all variables in table except poverty

*** Adjusted for age, sex, area and education

p-value for test of heterogeneity

**Table 5:** Vehicles involved in crashes causing traffic injuries in the urban and rural area

**Type of vehicle**	**Dar es Salaam (n = 68)**	**Hai (n = 23)**
	**%**	**%**
Car/truck	46	26
Bus	22	9
Bicycle	16	52
Motorcycle	13	4
Train	3	-
Cart	-	9

Our results show that rural residents were 1.6 times as likely as urban dwellers to experience a fall resulting in an injury. Falls were more likely in children aged below 15 years and adults 45 years and above (Table 4). They were reported to occur mainly in and around homes in the urban area (Table 6). In the rural area, outside home, on the roads and farms were reported to be the most frequent places of occurrence for falls.

**Table 6:** Place of injury by area and cause

**Place of injury**	**All injuries**		**Falls**		**Cuts**	
			
	**Urban (n = 206)**	**Rural (n = 303)**	**Urban (n = 56)**	**Rural (n = 83)**	**Urban (n = 38)**	**Rural (n = 149)**
	%	%	%	%	%	%
Home						
Inside	16.9	12.5	23.2	6.0	13.2	8.1
Outside	24.3	24.8	41.1	31.3	39.5	24.8
Workplace/factory	8.3	2.3	3.6	3.6	23.7	1.3
Farm	0	33.3	0	22.9	0	53.0
On the road	38.8	19.5	5.4	25.3	15.8	6.7
Recreation area including sports	9.7	1.9	21.4	2.4	7.9	1.3
School	1.9	4.6	5.4	8.4	0	4.0

Table 4 shows that rural inhabitants had a four fold risk of experiencing injuries due to cuts or stabs compared to urban residents (OR = 4.27; 95% CI = 2.96 – 6.15). It was noted that 68% (102/149) of injuries due to cuts in the rural area were related to agricultural activities, of which 81% occurred in adults aged 15 years and above. The farm and outside homes were where the injuries occurred most (Table 6). In the rural area, about 50% of children aged 5–14 years were injured when working on farms or around their homes and one third of the injuries were related to play. In the urban area, most (70%) of the children aged 5–14 years were injured while playing.

Burns accounted for about 6% (30/509) of all injuries in both areas. Children aged less than five years were 8 times as likely to sustain injuries due to burns as adults aged 15 to 44 years (OR = 8.58; 95% CI = 1.73 – 42.5). Although the magnitude of association appears to be large, the estimate was based on small numbers (16 and 5 injuries respectively; table not shown).

## Discussion

In this study, we found major differences between urban and rural residents with respect to cause and severity of injury and the circumstances in which they occurred. This has great implications in setting priorities when planning for intervention strategies.

Transport injuries formed the most common injury category in the urban area. The low risk of transport injury in the rural areas is probably a reflection of the relatively lower level of motorization in this mainly agricultural area. However, they will often have more serious consequences than other types of injury. A number of studies have reported similar findings. In a study from Pakistan, farmers were found to be at a lower risk of traffic injury than labourers and vendors [11]. A study from Bangladesh found a low incidence of traffic injury in a rural population [14]. Our data revealed that in the rural area, bicycle injuries predominated while in the urban area motorized vehicles accounted for a large proportion of transport injuries. Bicycles play a very important role in rural areas of Tanzania as a means of transport. In the urban area, most of the transport injury victims were passengers on public transport and pedestrians. Previous studies from developing countries have also reported the dominance of pedestrians, passengers of commercial vehicles and cyclists as vulnerable road users to transport injuries [10,11,21]. A hospital-based study conducted in an urban area in Tanzania reported that 42% of those subjected to transport injuries were pedestrians [4]. Strategies for prevention of transport-related injuries should take into account the local patterns.

Cuts and stabs by instruments such as axes and machetes constituted the most frequent injury category in the rural area, which is due to the fact that rural residents engage in agricultural activities using unprotected equipment. Cuts and stabs also contributed significantly among children aged 5 to 14 years with farm work being the common activity in the rural setting while play was the main contributing factor in the urban area. As emphasized in other studies, there is a need for safe space for play among children. In addition, the issue of a working child in developing countries like Tanzania needs to be addressed.

Falls were also a significant contributor among young children and older adults. A better understanding of the circumstances in which falls occur would assist in planning for fall-prevention programmes in Tanzania. Injuries due to accidental poisoning were infrequently reported in both areas and hence grouped under 'others'. It is possible that people were not comfortable reporting such events. In addition, near drowning did not feature among the causes of nonfatal injuries although the urban area has a port.

Except for transport injuries which were the commonest cause of fatal and nonfatal injuries in the same surveillance areas, the distribution of nonfatal injuries differed essentially from those of fatal injuries reported for 1992–1998 in the same surveillance areas [22]. In addition to transport injuries, the commonest causes of injury death were suicide, assault, accidental poisoning and drowning while the main causes of nonfatal injuries were falls, cuts and burns in these settings.

A significant risk factor for injury turned out to be the place of residence. The likelihood of self reported injury was 66% higher for rural residents compared to urban residents. Similar findings have been reported from other studies [13,23]. However, a study from Uganda found a high incidence of injury in the urban setting [12]. For severe injuries, we observed similar rates in the urban and rural areas. The main explanation is that transport injuries are more common and more severe in the urban areas whereas cuts and stabs are less common but more severe than in the rural area.

Age is an important risk factor for many injuries but its influence varies between specific injury groups. Our findings show that adults aged 15–44 years are at a high risk of transport injuries. This has great economic impact since these are people in their most productive years and the injuries impose a considerable burden on their families and the society as a whole. Children below 15 years were at greater risk of injuries due to falls. This may be due to high risk environments such as lack of proper play facilities. This is in keeping with studies from other developing countries whereby falls among children have been reported to be a common cause of injury seen in hospitals [24]. As expected, males were found to have an increased overall risk of injury which was more pronounced for transport injuries. A possible explanation may be that men spend more time on the roads than females and therefore they are more prone to high risk behaviours or unsafe road practices [25].

Socio-economic status has been documented to be an important determinant of injury, although the effect depends on the socio-economic indicator considered, the cause and severity of injury [7]. We found no significant relationship between income poverty and nonfatal injuries. This is consistent with findings from other studies [26,27]. Persons with primary education were at a greater risk of injuries than those with no formal education. This finding conforms to results from a previous study done in India [28].

The findings of this study are subject to a number of limitations. The information is based on self-reported data elicited through interviews, which is subject to recall bias [19,29]. A 12 month recall period was used in this study in order to include as many injuries as possible. Although long recall periods underestimate injury rates, they can be useful in investigating associations between injuries and different risk factors. Initial multiple logistic regression analysis was done using injuries reported in the three months prior to the interview only. We found that the pattern of the associations was similar to that based on all injuries except for area of residence where the size of the effect was stronger when a short recall period was used (results not shown). Other studies have demonstrated that relative risks are not affected when a long recall period is employed [30]. In a previous report, we found that memory decay was greater in the rural area than in the urban area [19]. Therefore, the actual difference in rates between the urban and rural area may have been underestimated.

Intentional injuries such as assaults and domestic violence are probably underreported since they would not be adequately captured in such a survey. This may lead to injury rates being underestimated. In this study, a clinical injury severity assessment was not possible. Disability days were used instead as a measure of severity of injury. One should be careful in generalizing the findings to other urban and rural settings of the country. Dar es Salaam is in many ways different from other urban areas of Tanzania and Hai is a relatively wealthy rural area. Furthermore, it might be difficult to generalize the findings to the surveillance areas due to the selection process that was employed. However, from our knowledge of the areas, we see no obvious reasons indicating that our samples should be very different from the urban and rural surveillance areas. Despite its limitations, this study has generated information that could be useful for targeted prevention at the local level.

## Conclusions

This study, the first of its kind in Tanzania, describes the patterns of nonfatal injuries and associated socio-demographic and socio-economic factors. It has attempted to identify specific groups of individuals as having a greater risk of experiencing certain types of injuries. This information is important for raising the level of awareness among policy makers and the public in general since the problem of injury receives little attention in most of the developing world including Tanzania. It is also useful in setting priorities for cause-specific prevention strategies. More detailed qualitative studies are required, however, on sensitive events such as assault. A nationally representative sample is also essential to measure the health burden due to nonfatal injuries.

## Competing interests

The author(s) declare that they have no competing interests.

## Authors' contributions

CM designed and conducted the study, performed statistical analysis, wrote the initial draft and revisions of the manuscript after consultation with other authors. IH, AN and GK participated in the design of study and revision of the manuscript. PS and YH participated in study design and co-ordination, and in revision of manuscript. All authors read and approved the final manuscript.

## Pre-publication history

The pre-publication history for this paper can be accessed here:


